# Single-Shot Structured Light Sensor for 3D Dense and Dynamic Reconstruction

**DOI:** 10.3390/s20041094

**Published:** 2020-02-17

**Authors:** Feifei Gu, Zhan Song, Zilong Zhao

**Affiliations:** 1 Shenzhen Institutes of Advanced Technology, Chinese Academy of Sciences, Shenzhen 518055, China; ff.gu@siat.ac.cn (F.G.); zl.zhao@siat.ac.cn (Z.Z.); 2 Mechanical and Automation Engineering Department, The Chinese University of Hong Kong, Hong Kong, China

**Keywords:** structured light, speckle, single-shot, dense reconstruction

## Abstract

Structured light (SL) has a trade-off between acquisition time and spatial resolution. Temporally coded SL can produce a 3D reconstruction with high density, yet it is not applicable to dynamic reconstruction. On the contrary, spatially coded SL works with a single shot, but it can only achieve sparse reconstruction. This paper aims to achieve accurate 3D dense and dynamic reconstruction at the same time. A speckle-based SL sensor is presented, which consists of two cameras and a diffractive optical element (DOE) projector. The two cameras record images synchronously. First, a speckle pattern was elaborately designed and projected. Second, a high-accuracy calibration method was proposed to calibrate the system; meanwhile, the stereo images were accurately aligned by developing an optimized epipolar rectification algorithm. Then, an improved semi-global matching (SGM) algorithm was proposed to improve the correctness of the stereo matching, through which a high-quality depth map was achieved. Finally, dense point clouds could be recovered from the depth map. The DOE projector was designed with a size of 8 mm × 8 mm. The baseline between stereo cameras was controlled to be below 50 mm. Experimental results validated the effectiveness of the proposed algorithm. Compared with some other single-shot 3D systems, our system displayed a better performance. At close range, such as 0.4 m, our system could achieve submillimeter accuracy.

## 1. Introduction

Three-dimensional (3D) dynamic reconstruction is an important technique in the field of computer vision that has been widely applied in manufacturing, industrial inspection, human–computer interaction, robotics, augmented reality (AR)/virtual reality (VR), and so on [1,2]. Currently, 3D dynamic reconstruction techniques mainly include time of flight (TOF) [3,4], binocular stereo vision (BSV) [5,6], and structured light (SL) [7,8]. All these techniques have advantages and disadvantages, which are as follows: (1) TOF technique: The TOF technique uses active sensing to capture 3D range data as per-pixel depths [9]. A near-infrared wave is emitted by the light source from the camera, and then its reflected wave is recorded by a dedicated sensor. By measuring the time delay, the distance at each pixel can be estimated. The TOF technique has the advantages of high integration and a fast response time; however, it has the disadvantages of low depth resolution and more noise when working in close range. (2) Binocular stereo vision (BSV) technique [10]: BSV is referred to as a passive vision system that recovers 3D point clouds via the triangulation structure constituted by two cameras. It first requires detecting and matching feature points from the captured stereo images, and therefore can only conduct sparse reconstruction. It cannot measure objects with poor textures. (3) Structured light (SL) technique [11]: SL also works on the principle of triangulation, except that an active light projection source is added. Compared with a passive vision system, it straightforwardly addresses the stereo matching problem and enhances the performance in measuring textureless regions. The SL technique can be divided into temporally coded SL [12] and spatially coded SL [13] according to different codification strategies. Temporally coded SL projects a sequence of patterns onto the object surface, and 3D surface information can be recovered when all patterns are gathered together. Spatially coded SL only demands a single shot, in which the codeword of each pattern feature is determined using its own values, along with those of all adjacent elements surrounding it. Gray level [14], color [15], and geometric shapes [16] are the most frequently adopted pattern element types in spatially coded SL.

As outlined above, SL outperforms TOF due to its higher resolution and less noise and outperforms BSV when handling textureless surfaces. Nevertheless, SL techniques have a trade-off between acquisition time and spatial resolution. Temporally coded SL can obtain dense point clouds, but it is not suitable for dynamic reconstruction; on the contrary, spatially coded SL can work in real time, but it can only recover sparse point clouds. To conduct 3D dense reconstruction and 3D dynamic reconstruction, this study developed a single-shot SL based on pseudo-random speckles. The proposed system consists of two cameras and a projector. An infrared light speckle pattern is projected to attach textures onto object surfaces, and the reflected light is captured by two infrared sensors. Key steps of the proposed system include system calibration, epipolar rectification, and stereo matching. First, both cameras should be calibrated and rectified, then depth information can be recovered by conducting stereo matching between left and right images along the same epipolar lines. 

The core problem with speckle-based SL is stereo matching. The task of stereo matching is to find an accurate corresponding pixel in the right image for each pixel of the left camera. Accurate stereo matching is the premise of accurate measurement. Though there is much mature research in this area, most methods cannot work well enough when directly applied to speckle images. Stereo matching algorithms are generally divided into local and global methods [17]. Typical local methods are performed in four steps: matching cost computation, cost aggregation, disparity computation, and disparity refinement [18]. One representative local method is block matching (BM) [19]. In computing the matching cost, a support window is centered on every pixel of the stereo image to find corresponding points with the lowest intensity dissimilarity. Though local methods work fast, they have an implicit assumption that all pixels in the support window have a constant disparity, which leads to an estimation error in measuring slanted surfaces. Furthermore, the size of the support window is important; a smaller window brings higher noise, while a larger window may over-smooth local details and depth boundaries [20]. Global methods skip the step of cost aggregation and achieve optimal disparity by finding the minimum of a global energy function. By adding a smoothness term in the global energy function, a better and smoother disparity map can be achieved. However, most of the current top-ranked global methods have a high complexity and long run times [21,22]. Considering both running efficiency and finer disparity maps, the semi-global matching (SGM) method [23] has been preferred for speckle-based SL. Usually, SGM works well in most cases; however, it also has the local method disadvantage of a constant disparity assumption in the procedure of calculating matching costs.

The contributions of this paper are as follows. First, the distribution of pseudo-random speckles is specifically designed to provide local features with rich textures and high identification. Then, an accurate camera calibration and epipolar rectification algorithm are proposed. Lastly, an optimized semi-global matching (SGM) algorithm is proposed to improve the correctness of the stereo matching. The paper is organized as follows: Section 2 describes the speckle pattern design, camera calibration, epipolar rectification, and stereo matching algorithms adopted in the proposed SL. The experimental results are given in Section 3. Conclusions are presented in Section 4. 

## 2. Key Technology and Algorithm

**Figure 1:**
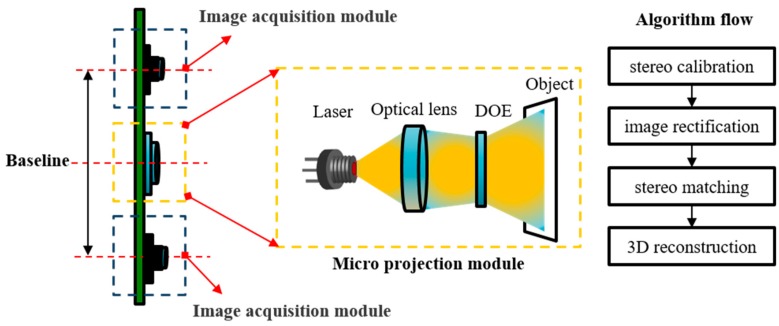
Schematic diagram of proposed structured light system. DOE: diffractive optical element.

Most structured light systems rely on expensive and high-powered projection devices. To reduce the system cost, a diffractive optical element (DOE) projector was utilized in our structured light system. The schematic diagram of our proposed system is displayed in Figure 1. The detailed procedures of our algorithm are described below.

### 2.1. Design of the Speckle Pattern

The speckle pattern is projected onto the object’s surface to provide rich textures; therefore, the first step is to design a good pattern. Preferably, pseudo-random dots are utilized [24]. The random distribution characteristics of speckle dots form the prerequisite and foundation for unique feature matching. To further assist robust and accurate feature matching, the design of the speckle distribution should meet one basic requirement: rich textures. 

**Figure 2:**
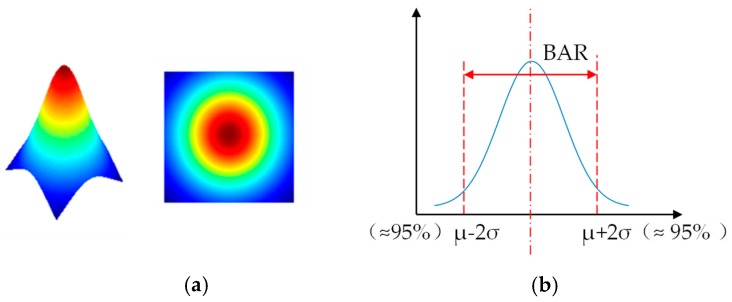

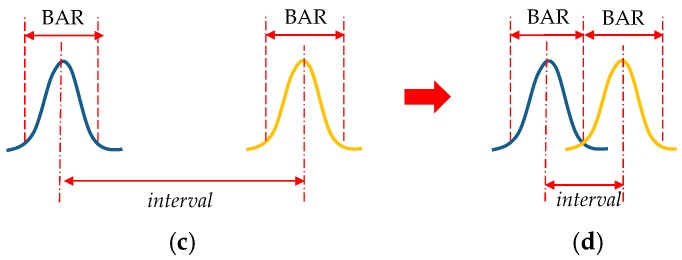
Definitions of the brightness attenuation range (BAR) and interval of projected dots: (**a**) brightness distribution of optical dots, (**b**) definition of the BAR, (**c**) definition of the interval between two adjacent dots, and (**d**) optimal interval.

Before elaborating on the method of designing a rich-textured speckle pattern, two important definitions should be introduced first. The distance between adjacent speckle dots, called an interval, is a key index that determines the texture richness. The value of the interval is closely related to the brightness attenuation range (BAR) of each projected dot. The definitions of the interval and BAR are illustrated in Figure 2. Ideally, the brightness distribution of optical dots conforms to a Gaussian distribution, as shown in Figure 2a. The BAR is generally defined as the pixels where the brightness is reduced by 95%, which is displayed in Figure 2b. In practice, the actual BAR of the projected dots is independent of the speckle pattern itself but is affected by two main factors: (a) the optical design and engineering capability in manufacturing the DOE projector, and (b) the projection distance. Therefore, being affected by different processing levels and working distances of the DOE projector means that different BARs may occur. In this case, the actual BAR can only be empirically determined, which can take the mean BAR as a reference. 

As can be seen from Figure 2c, the interval is a key index that determines the texture richness. If the interval is too large, the distribution of dots is sparse, which may lead to less texture; if the interval is too small, the distribution is denser, yet it may lead to more overexposure areas with unrecognizable textures. Therefore, it is important to choose an appropriate interval. It is not hard to see that one of the most reasonable and convenient ways is to set the value of the interval equal to the value of the BAR, as shown in Figure 2d.

Now we return to the question of designing a good pattern. To make use of the above-mentioned observation, an algorithm to generate a pseudo-random speckle pattern and a predefined constraint window was proposed and illustrated with pseudo-code, as shown in Algorithm 1. In practice, the size of the constraint widow is set according to the BAR of each projected dot.
**Algorithm 1. Generation of a Speckle Pattern***// pre-define the constraint window*// set the resolution of speckle pattern: width * height**For** loop = 1:height * width, **do**  **u**: generate a random row coordinate within the range of {1, *height*}  **v**: generate a random column coordinate within the range of {1, *width*}  // *Judge whether the constraint condition is satisfied or not*  **If** there are no dots in the constraint window centered at (**u**,**v**)    Put a dot at (**u**,**v**)  **Else**    No dots are added  **End If****End For**

**Figure 3:**
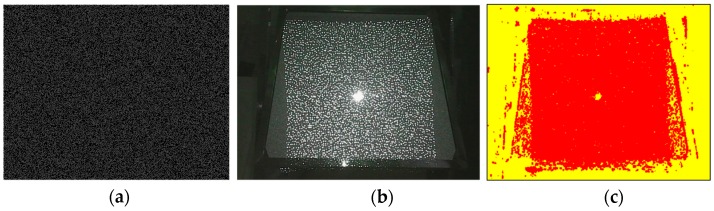
Speckle pattern designed in this paper: (**a**) designed speckle pattern, (**b**) speckle pattern projected using DOE, and (**c**) richness of the textured regions; red represents textured regions and yellow represents textureless regions.

In this paper, we designed a pattern with a resolution of 640 × 480. Before designing the speckle pattern, we learned the empirical BAR value from the manufacturer, which helped to conduct multiple simulations and machining tests before the final version. At the current machining level, the BAR was about 5 pixels. Thus, the predefined constraint window was set at 5 × 5 pixels. The designed speckle pattern is displayed in Figure 3a. This pattern was then projected onto the surface of a standard plane using a DOE projector, as shown in Figure 3b. It can be seen that most speckle dots could be projected correctly. The overexposure areas at the center of the pattern were caused by the projection device, which is known as the zero-order diffraction phenomenon. It can be eliminated by improving the optical design of the microprojection module, which will not be further discussed here since it is not the focus of this paper. The texture richness of the designed pattern was evaluated by using the method described in Appendix A and the results are shown in Figure 3c. The textured regions are marked by red dots, and the textureless regions are marked by yellow dots. It can be seen that most regions covered by the projected pattern are textured.

After projecting the speckle pattern onto the target surface, the two cameras shown in Figure 1 captured the textured images. In speckle-based SL, the primary problems most likely occur in the processes of camera calibration, epipolar rectification, and stereo matching; therefore, we will elaborate on these three problems.

### 2.2. Epipolar Rectification

**Figure 4:**
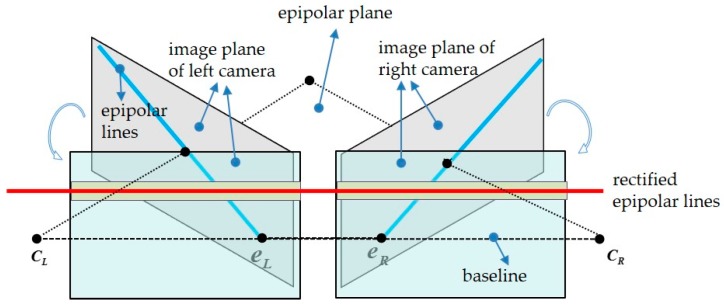
Schematic diagram of stereo rectification.

Given the fact that the two cameras in speckle-based SL cannot be strictly aligned in the process of assembly, stereo rectification is necessary before stereo matching. The transformation of a stereo rig before and after epipolar rectification is illustrated in Figure 4. Taking the left camera coordinate system (*CCS_L_*) as the reference coordinate system, before rectification, the relative transformation of the right camera coordinate system (*CCS_R_*) against *CCS_L_* is [*R T*]. To make all epipolar lines parallel to assist feature matching, the rectified *CCS_L_* and *CCS_R_* should be horizontally aligned, which means there is no rotation, but only a translation $T^{rec}$ exists between them.

**Figure 5:**
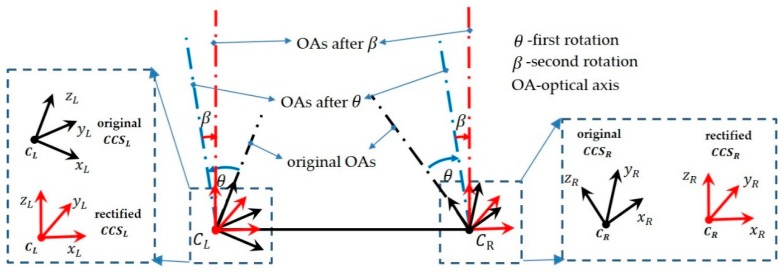
Stereo rectification by two rotations. CCS: camera coordinate system.

In this paper, we put forward an efficient epipolar rectification method based on two rotations of both cameras. The detailed procedure is displayed in Figure 5.

First rotation: Assume the original angle between the left optical axis (OA) and the right OA is *2θ*. To minimally rotate both cameras such that they have the same orientation, rotate each camera with an angle of *θ* in opposite directions. This is exactly half of the current rotation relationship R between the two cameras. In order to relate R and *θ* directly, the rotation vector om was introduced. om, a 3 × 1 vector, is a more compact way to describe R. The direction of om is the rotation axis and the length of om is the rotation angle around the rotation axis. Therefore θ=‖om‖/2. In this way, the rotation matrix of the left camera can be determined according to Rodrigues’s equation:

(1) $$R_{L}^{\theta }=\cos(\theta )\cdot I+(1-\cos(\theta )){\left[\omega \right]}_{\times }^{2}+\sin(\theta ){\left[\omega \right]}_{\times }.$$

In Equation (1), ${\left[\cdot \right]}_{\times }$ denotes the antisymmetric matrix. The rotation axis is denoted by ω: ω=om/θ. I is a 3-order identity matrix. The rotation matrix of the left camera is in the opposite direction, which can be determined using $R_{R}^{\theta }={\left(R_{L}^{\theta }\right)}^{-1}$. Through the first rotation, the left camera and right camera are brought to the same orientation, as shown by the blue dotted lines in Figure 5. The translation vector after the first rotation is:

(2) $$T^{\theta }=R_{R}^{\theta }\cdot T.$$

Second rotation: After the first rotation, the new OAs are parallel, yet they are not perpendicular with the axis ζ constituted by optical centers $C_{L}$ and $C_{R}$. Therefore, a common rotation $R^{\beta }$ for both cameras was necessary. Denote $\zeta ={\left[1,0,0\right]}^{T}$. Therefore, a second rotation was required to bring the translation vector in alignment with ζ. The direction of the new rotation axis ξ can be calculated using ζ and $T^{\theta }$:

(3) $$\xi =(T^{\theta }\times \zeta )/\left\|T^{\theta }\times \zeta \right\|.$$

The rotation angle around ξ can be determined using:

(4) $$\beta =\arccos[(T^{\theta }\cdot \zeta )/(\left\|T^{\theta }\right\|\left\|\zeta \right\|)].$$

Here, arccos[·] denotes the inverse cosine. Then, the rotation axis in the second rotation can be uniquely determined using ξ=ξ⋅β. By using the same equation as in Equation (1), the second rotation $R^{\beta }$ can be obtained. 

Combining the above two rotations, the renewed rotation and translation between the left and right cameras after stereo rectification are obtained as follows:

(5) $$\begin{array}{c} R_{R}^{rec}=R^{\beta }\cdot R_{R}^{\theta }\\ R_{L}^{rec}=R^{\beta }\cdot R_{L}^{\theta }\\ T^{rec}=R^{\beta }\cdot T^{\theta }=R^{\beta }\cdot R_{R}^{\theta }\cdot T \end{array}.$$

Rectified intrinsic parameters: To get the rectified focal length, lens distortions of the camera should be considered. The common model can be generalized as:

(6) $$x=x_{d}+\left[{\bar{x}}_{d}(k_{1}r_{d}^{2}+k_{1}r_{d}^{4})+(2p_{1}{\bar{x}}_{d}{\bar{y}}_{d}+p_{2}(r_{d}^{2}+2{\bar{x}}_{d}^{2}))\right].$$

Here, $\left(x_{d},y_{d}\right)$ are the distorted pixel coordinates and $\left(u_{0},v_{0}\right)$ is the imaging center. Furthermore, ${\bar{x}}_{d}=(u_{d}-u_{0})/f_{x}$, ${\bar{y}}_{d}=(v_{d}-v_{0})/f_{y}$, and $r_{d}=\sqrt{{\bar{x}}_{d}^{2}+{\bar{y}}_{d}^{2}}$. $\left(k_{1},k_{2}\right)$ and $\left(p_{1},p_{2}\right)$ are the coefficients of the radial and tangential distortions, respectively. To eliminate the effects of lens distortion, the rectified focal length is defined as:

(7) $$f_{x}^{rec}=f_{x}+\left[f_{x}(k_{1}{ℜ}^{2}+k_{1}{ℜ}^{4})+(2p_{1}{\bar{ℜ}}_{x}{\bar{ℜ}}_{y}+p_{2}({ℜ}^{2}+2{\bar{ℜ}}_{x}^{2}))\right].$$

Assume the image resolution of the camera is W×H. In Equation (7), ${\bar{ℜ}}_{x}=H/2$, ${\bar{ℜ}}_{y}=W/2$, and $ℜ=\sqrt{{\bar{ℜ}}_{x}^{2}+{\bar{ℜ}}_{y}^{2}}$. $f_{x}$ is the original focal length. The rectified focal length of the left and right cameras is set the same: $f^{rec}=[f_{x}^{rec},f_{x}^{rec}]$.

**Figure 6:**
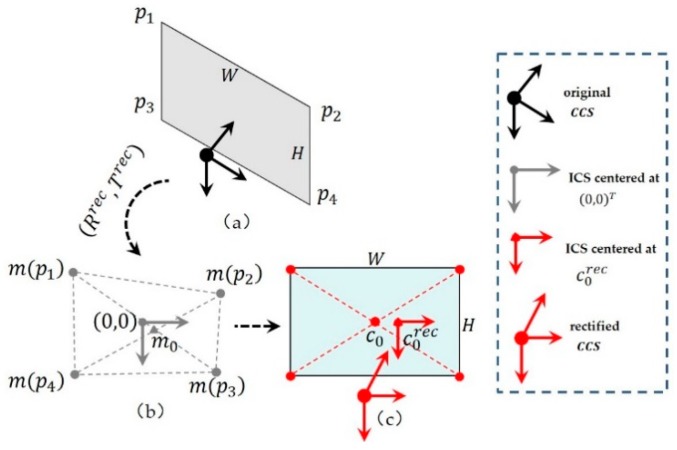
Determination of the imaging center after stereo rectification: (**a**) four vertices of the image plane in original *CCS*, (**b**) assumed image coordinate system (*ICS*) centered at ${\left(0,0\right)}^{T}$, and (**c**) rectified *CCS* and *ICS*.

As for the imaging center, the average value of both cameras’ imaging centers was generally taken as the rectified cameras’ imaging centers, which is inaccurate in practice [25]. In fact, rectified images will be twisted or rotated more or less compared with original images. The positions of imaging centers will also likely affect the sizes of visible areas in rectified images. With other parameters being determined (extrinsic parameters, focal length, etc.), the rectified image centers can be better located based on known parameters. The detailed procedure is illustrated in Figure 6.

First, as shown in Figure 6a, four vertices $\left\{p_{1},p_{2},p_{3},p_{4}\right\}$ of the original image plane are projected into the rectified CCS by assuming that the rectified imaging center is at ${\left(0,0\right)}^{T}$:

(8) $$m_{0}=\sum_{i=1}^{4}K_{\rho }^{rec}\cdot [R_{\rho }^{rec}\;T_{\rho }^{rec}]\cdot p_{i}/4(\rho =L\;or\;R).$$

Here, $K^{rec}=\left[\begin{array}{ccc} f_{x}^{rec} & 0 & 0\\ 0 & f_{x}^{rec} & 0\\ 0 & 0 & 1 \end{array}\right]$. For the left camera (ρ=L), $T_{\rho }^{rec}={\left[0\;0\;1\right]}^{T}$; for the right camera (ρ=R), and the value of $T_{\rho }^{rec}$ refers to Equation (5). The overall imaging area can be achieved as shown in Figure 6b, with the image coordinate system (*ICS*) centered at ${\left(0,0\right)}^{T}$. Generally, the rectified area is of a twisted and irregular shape. 

Second, the rectified area in Figure 6b is fit into the image plane of size W×H as displayed in Figure 6c. To maximize the visible area of Figure 6b in Figure 6c, the renewed imaging center $c_{0}^{rec}$ after rectification can be adjusted using:

(9) $$c_{0}^{rec}=c_{0}+m_{0}.$$

Here, $c_{0}={\left[W/2,H/2\right]}^{T}$. Then, the renewed *ICS* and *CCS* after the epipolar rectification can be built as shown in Figure 6c. 

After obtaining the rectified intrinsic and extrinsic parameters of the two cameras, epipolar rectification can be accomplished by nonlinearly mapping original images into the rectified *ICS*. With both left and right images rectified, feature matching can be conducted along the direction of the image rows, which reduces the complexity of matching and speeds up the matching process.

The advantage of the above rectification method is obvious. First, both cameras rotate around the same angle in each step of the transformation, which can minimize feature differences between the left and right images. Second, the rectified images keep the same resolution with the original images, which is very convenient in stereo matching because most of the accessible dense matching methods require the same size left and right images. Lastly, the maximal visible image area can be guaranteed in the rectified image, which can minimize image information loss during the stereo rectification process.

### 2.3. Stereo Calibration

It is not hard to see from Section 2.1 that the accuracy of epipolar rectification is closely related to the accuracy of the stereo calibration. However, traditional camera calibration methods do not consider the accuracy of epipolar rectification; most of them focused on estimating the optimal stereo parameters that can minimize 2D re-reprojection errors [26] or 3D reconstruction errors [27]. Among them, Zhang’s method [26] has been widely used as a flexible camera calibration method that takes 2D reprojection errors as the cost function. In practice, a slim misalignment of epipolar lines may lead to large errors in feature matching, which will inevitably hinder accurate reconstruction. 

In our previous work [27], an accurate camera calibration method was proposed based on the backprojection process, in which 3D reconstruction errors were designated as the cost function to improve the 3D reconstruction accuracy of a stereo rig. Details can be found in Gu et al. [27]. In this paper, an additional term describing the epipolar distance errors was added in the cost function of the camera calibration, which is defined as:

(10) $$e_{epi}=\sqrt{d{\left(m_{R},Fm_{L}\right)}^{2}+d{\left(m_{L},F^{T}m_{R}\right)}^{2}}.$$

Here, d(α,η) denotes the distance from point α to line η. The fundamental matrix F can be uniquely determined once calibration is done. $\left(m_{L},m_{R}\right)$ is a pair of matching points of the left and right images. Then, the new cost function E(x) was redefined as:

(11) $$E(x)=E_{3D}(x)+\alpha E_{epi}(x).$$

Here, $E_{3D}(x)$ denotes the term representing 3D reconstruction errors and $E_{epi}(x)=\sum e_{epi}$. α is a coefficient that adjusts the weight of the two cost terms, which is set to 0.5 in practice. By minimizing E(x), optimal camera parameters guaranteeing minimal 3D reconstruction errors and epipolar distance errors can be achieved.

### 2.4. Stereo Matching Based on an Improved SGM

According to Section 2.2 and Section 2.3, feature matching can be conducted along the rectified epipolar lines, which now remain in accordance with the direction of the image rows. Accurate feature matching has a pivotal role in accurate 3D reconstruction. The SGM algorithm achieves a good balance between high-quality depth maps and running efficiency [23]. Besides the pixel cost calculation, additional constraints are added in the energy function E(D) to support smoothness, as shown in Equation (12). The problem of stereo matching is to find an optimal disparity map *D* that can minimize the energy E(D):

(12) $$E(D)=\sum_{p}(\mathrm{Cos}t(p,d)+\sum_{q\in N_{p}}V(d_{p},d_{q})).$$

In Equation (12), the first term is the sum of all pixel-matching costs of the disparities in *D*. The second term adds constant penalties for all pixels q in a neighborhood $N_{p}$ of p. This extra term penalizes changes in neighboring disparities. One characteristic of SGM the aggregation of matching costs of 1D from all directions equally. Along each direction r, the cost of pixel p at disparity d is defined recursively as:

(13) $$L_{r}(p,d)=Cost(p,d)+\min\{\begin{array}{c} L_{r}(p-r,d)+V(d_{p},d_{p-r})\\ L_{r}(p-r,d-1)+V(d_{p},d_{p-r})\\ L_{r}(p-r,d+1)+V(d_{p},d_{p-r})\\ \min_{i}L_{r}(p-r,i)+V(d_{p},d_{p-r}) \end{array}.$$

The first term is the calculated cost of disparity d. The second term is the lowest cost of the previous pixel of p−r along the path. $V(d_{p},d_{p-r})$ is a penalty term that penalizes changes in neighboring disparities. It is defined by Equation (14). The second term in Equation (12) can be specifically interpreted as follows: If the disparity in pixel p−r stays the same as the disparity in p, no penalty is added. If the disparity in pixel q changes only one pixel, the penalty factor $P_{1}$ is utilized. For larger disparity changes (that is, greater than one pixel), the penalty factor $P_{2}$ is added. Generally, pixels with larger disparity changes have little effect on the final results; therefore, $P_{2}$ is generally set to a large value.

(14) $$V(d_{p},d_{p-r})=\{\begin{array}{cc} 0 & if\;d_{p}-d_{p-r}=0\\ P_{1} & if\;|d_{p}-d_{p-r}|=1\\ P_{2} & if\;|d_{p}-d_{p-r}|>1 \end{array}$$

Recent works have found that SGM has the same assumption as local matching methods, namely that all pixels in the matching window have constant disparities. This implicit assumption does not hold for slanted surfaces and leads to a bias toward reconstructing frontoparallel surfaces. The famous PatchMatch method proposed by Bleyer [20] tries to overcome this problem by estimating an individual 3D plane at each pixel onto which the current matching block is projected. However, it inevitably increases the execution time, which impairs the real-time performance of the 3D vision system. 

**Figure 7:**
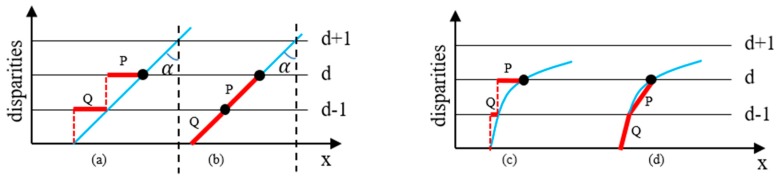
Disparity searching in 1D. Points of the blue surface are to be reconstructed. The disparity search path is shown by red bars. (**a**) Disparity estimation route of a plane with semi-global matching (SGM). (**b**) Ideal disparity estimation route of a plane. (**c**) Disparity estimation route of a curved surface with SGM. (**d**) Ideal disparity estimation route of a curved surface.

**Figure 8:**
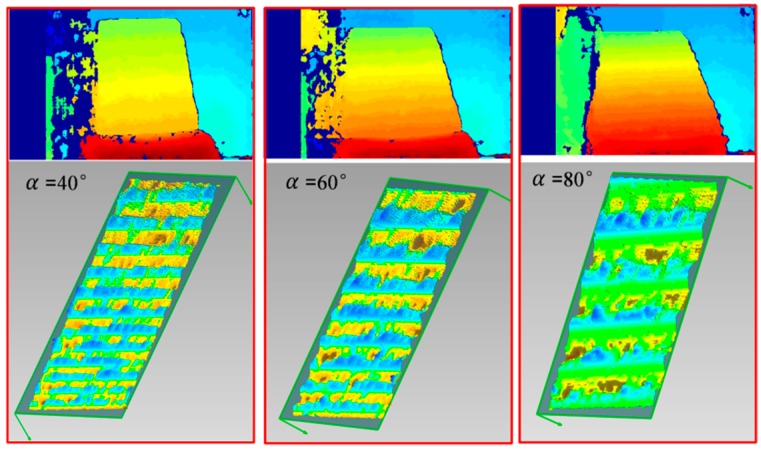
Ladder effect of SGM for slanted surfaces with different tilt angles.

We experimentally found that the penalty term $V(d_{p},d_{p-r})$ reinforced the above-mentioned assumption to a great extent. Figure 7 illustrates this problem. Take a slanted surface and a curved surface as examples. Ideal disparity estimation routes are displayed in Figure 7b,d as a comparison. However, in the traditional SGM algorithm, the case of keeping constant disparities is encouraged by giving a smaller penalty ($V(d_{p},d_{p-r})=0$) to the state of $d_{p}=d_{p-r}$. This inevitably leads to inaccurate disparity results, such as P and Q in Figure 7a,c. In fact, the penalty mechanism in Equation (13) only works well for frontoparallel surfaces ($\alpha =90^{{}^{\circ}}$ in Figure 7a) and does not hold for slanted or curved surfaces. In practice, it will lead to a ladder effect in reconstructing non-frontoparallel surfaces (refer to Figure 8). The ladder effect causes a perceptible change when the angle of α changes. It can be seen that with the increase of α, the ladder effect becomes gentler and gentler, which is in accordance with the analysis in Figure 7a.

Therefore, we adapted the traditional SGM algorithm to accommodate this new observation. In practice, a new penalty term is defined, as given in Equation (15), to alleviate this kind of bias. In this case, equal penalties were utilized for unchanged disparities and disparities that change only one pixel.

(15) $$V^{\prime }(d_{p},d_{p-r})=\{\begin{array}{cc} 0 & if\;|d_{p}-d_{p-r}|\leq 1\\ P_{2} & if\;|d_{p}-d_{p-r}|>1 \end{array}$$

Using the above improvement, the ladder phenomenon can be alleviated effectively. Detailed experiments are described in Section 4. The improved SGM plays an important role in improving the 3D measurement accuracy of speckle-basedSL. After stereo matching, 3D information can be achieved based on the triangular structure constituted by the two cameras in the system.

## 3. Experiments and Results

**Figure 9:**
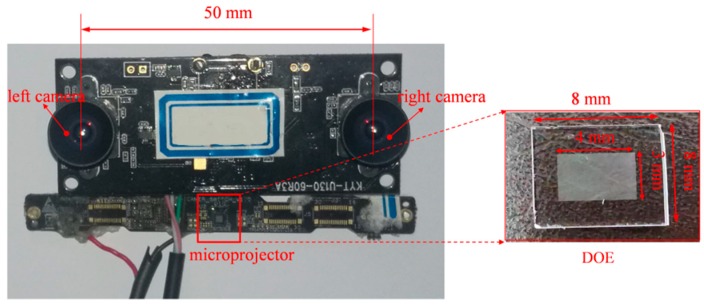
Experimental setup.

A prototype of our system is shown in Figure 9, which consisted of two cameras with a resolution of 1280 × 960 and a DOE projector with 12,000 pseudo-random speckle dots etched on it. The stereo module was customized and packaged by QuanRui (Shenzhen, China). It could capture stereo images synchronously at a frame rate of up to 60 fps. Automatic exposure control (AEC) and automatic white balance (AWB) were adopted. The DOE projector was designed to be 8 × 8 mm and had a field of view of 55° × 45°. The DOE device was attached to a laser source that worked at 850 nm with power of 0.2 W. The depth of focus of the DOE projector was 0.03 m to 1.6 m. The baseline of the system was about 50 mm, and an efficient working distance was 0.03 m to 1.60 m. To reduce the effect of ambient light, an IR-cutoff filter was attached to the camera lens. The proposed algorithms were implemented using VS2015 on a computer with an i7-9850H central processing unit and 32 GB of random access memory. After system calibration, several experiments were conducted on our proposed vision system. The first experiment was to test the performance of the proposed camera calibration algorithm. Then, the performance of the improved SGM algorithm was tested. Afterward, the measurement accuracy of the system was evaluated by comparing it with other common depth systems at different distances. Then, surfaces with rich colors and complex textures were selected to test the robustness of our system. Some dynamic targets, such as facial expression, waving an arm, and a working fan, were also tested.

### 3.1. System Calibration

Our SL system was calibrated and rectified based on the methods proposed in Section 2.2 and Section 2.3. The results are listed in Table 1. One of the rectification results of the calibration images is displayed in Figure 7 as an example.

**Figure 10:**
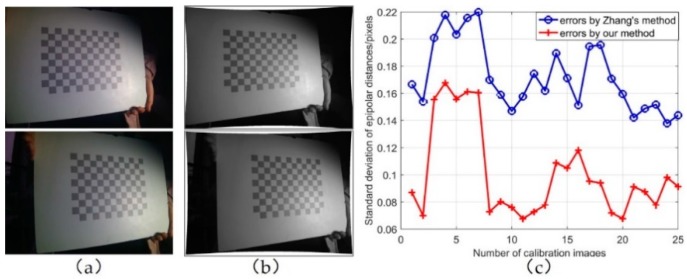
Stereo images before and after epipolar rectification: (**a**) original images, (**b**) rectified images using proposed method, and (**c**) accuracy comparison of proposed method with Zhang’s method.

In the experiments, 25 images were utilized for stereo calibration, each containing 108 points. The epipolar distance errors (EDEs) created using Zhang’s method [26] and our method were calculated and compared, as shown in Figure 10c. The EDE is defined in Equation (10).

In Zhang’s method, the mean value of EDE for all calibration images was 0.172 pixels; in our method, the mean value of EDE was 0.100 pixels. The accuracy was improved by up to 71.4%. 

### 3.2. Performance Evaluation of the Improved SGM Algorithm

**Figure 11:**
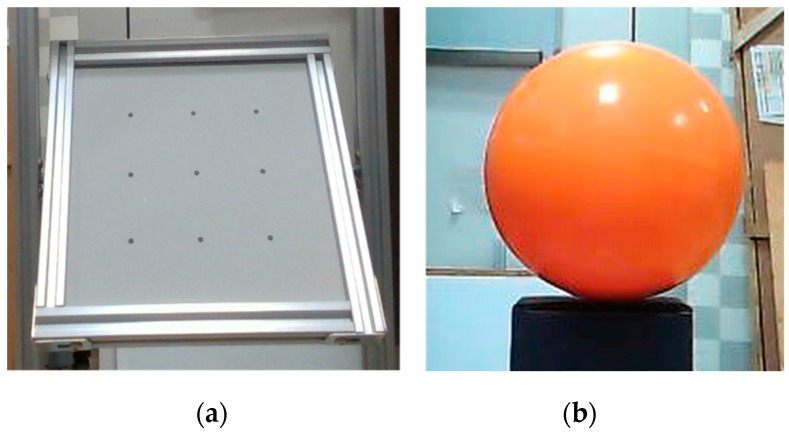
Reference objects for accuracy evaluation: (**a**) standard plane and (**b**) standard sphere.

To validate the effectiveness of the improved SGM, a standard plane and a sphere were utilized as the target objects, as displayed in Figure 11. The plane was 20 cm × 20 cm in size and its machining accuracy was up to ±5 μm. It was put at a tilted angle in the experiment. The sphere had a diameter of 15 cm and its machining accuracy was up to ±20 μm. 

**Figure 12:**
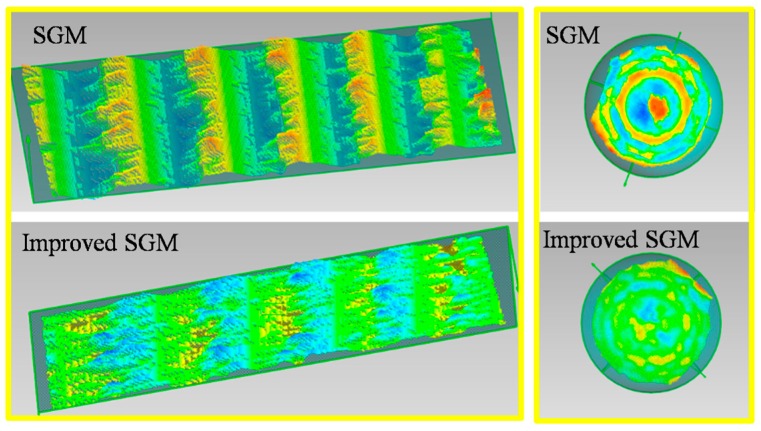
Effect comparison of SGM and improved SGM.

First, both surfaces were reconstructed using the SGM and the improved SGM. Without loss of generality, the parameters in both methods were set to be the same, such as the size of the matching window, and both methods used the same post-processing methods, which mainly included the uniqueness check and the left/right consistency check. The results are displayed in Figure 12. It can be seen that on both surfaces, the ladder effect could be effectively alleviated using our method. 

Second, the plane and the sphere ware put at different distances from our vision system to test the measurement accuracy of our algorithm. The plane-fitting error and sphere-fitting error were designated as the indices to evaluate the 3D reconstruction accuracy of the traditional SGM and our improved SGM. A comparison of the results is displayed in Table 2. At close range, such as 0.4 m, the measurement accuracy of our method was up to 0.553 mm for the 2D plane and 0.465 mm for the 3D sphere. The measurement errors were reduced by about 14.3% and 30.4%, respectively. At a distance of 1.0 m, our method performed even better. The measurement errors were reduced by 38.0% and 44.5% for the plane and the sphere, respectively.

### 3.3. Accuracy Comparison with Other Techniques

**Figure 13:**
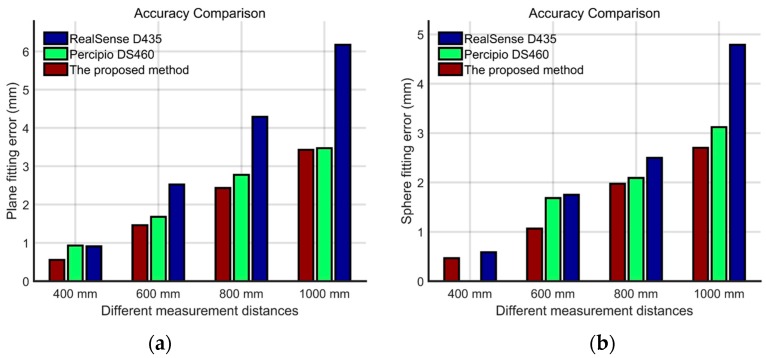
Accuracy comparison between D435, Kinect v2, and our system: (**a**) plane-fitting errors and (**b**) sphere-fitting errors.

**Figure 14:**
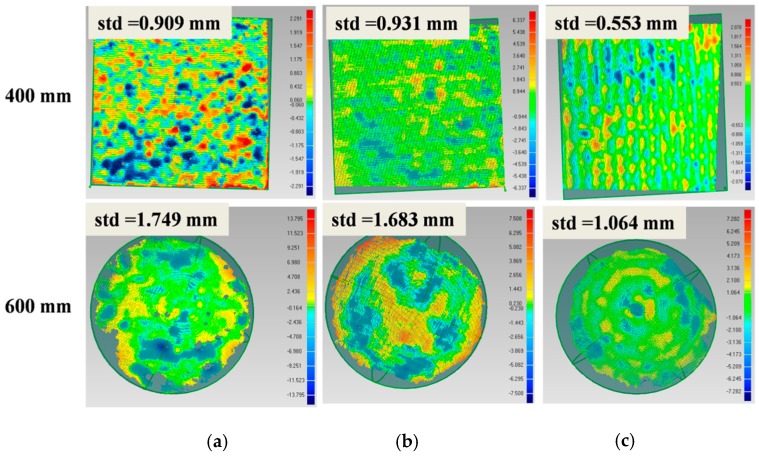
Accuracy comparison with other systems: (**a**) RealSense D435, (**b**) Kinect v2, and (**c**) the proposed system.

To further show the effectiveness of our low-cost vision system, two other common 3D depth cameras, Intel RealSense D435 and Microsoft Kinect v2, were utilized for comparison. The standard surfaces in Figure 11 were also used in this experiment. Results from different measurement distances (0.4 m, 0.6 m, 0.8 m, and 1.0 m) were compared, and are shown in Figure 13. To save some space, the results of the plane data at 0.4 m and the sphere data at 0.6 m were taken out and displayed in Figure 14.

Note that Kinect v2 does not work for close range, and therefore part of the plane and sphere at 0.4 m could not be measured; the corresponding data are missing in Figure 13b and Figure 14. Several conclusions can be drawn from Figure 13. First, the error-changing trends of RealSense D435 and our system in measuring both objects were very similar. As the distance increased, the error increased approximately linearly. This was due to the fact that they both work on the principle of triangulation. Second, our system achieved better accuracy than RealSense D435 at all distances because it used optimized algorithms for system calibration, epipolar rectification, and stereo matching, as introduced in Section 3. At the close range of 0.4 m, the plane’s measurement error of the proposed system was 0.553 mm, and the error of Intel RealSense D435 was 0.909 mm, which was 64.4% higher than our system. As for the sphere data, the measurement error of the proposed system was 0.465 mm, while the error of Intel RealSense D435 was 0.586 mm, which was 26.0% higher than our system. Third, Kinect v2 had a relatively stable measurement accuracy because it works on the principle of time-of-flight (TOF). Our system outperformed Kinect v2 at close range near 0.6 m, but gradually became inferior as the distance increased. This was determined by the characteristics of the triangle structure itself. It should be noted that at most of the distances, our system performed better than Kinect v2 at measuring the sphere because the TOF technique cannot handle the edges of objects well and it is easy to be affected by the different reflectivity of object surfaces. 

In summary, it is safe to say that the proposed system achieved a better accuracy than RealSense D435, and had a better adaptability than TOF techniques, such as that used in Kinect v2, which makes it a feasible solution for various 3D vision tasks, such as somatic games, gesture recognition, face unlocking, face payment, and so on. 

### 3.4. Reconstruction of Surfaces with Rich Textures

**Figure 15:**
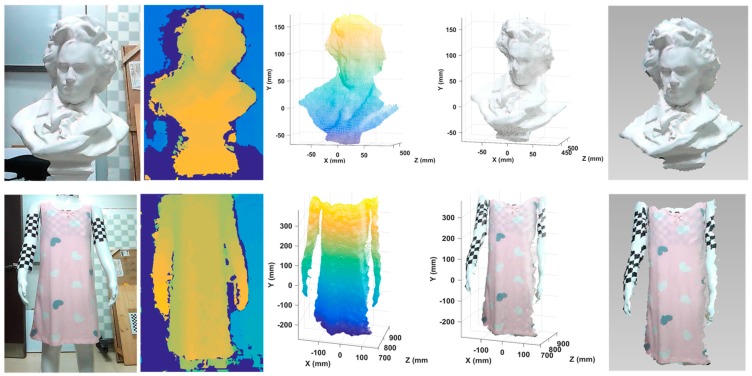
Reconstruction results of surfaces with rich textures using the proposed system. From left to right: original images, estimated depth maps, reconstructed point clouds, point clouds with textures, and 3D models.

In this part, experiments were conducted to test our system by reconstructing surfaces with rich textures. One was a sculpture of a head with rich geometric textures and another was a dress with rich color textures. Corresponding results, including estimated depth maps based on our improved SGM, point clouds based on triangulation, point clouds with textures, and 3D models, are displayed in Figure 15. It can be seen that our system worked well on complex surfaces. To offer a more intuitive visual effect, an extra digital single lens reflex (SLR) camera was utilized in this experiment to attach textures to 3D point clouds.

### 3.5. Reconstruction of Dynamic Objects

**Figure 16:**
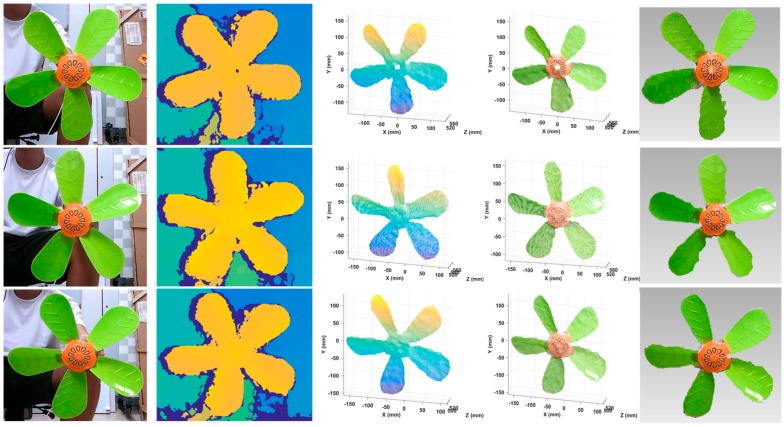
Reconstruction results of a working fan using the proposed system. From left to right: original images, estimated depth maps, reconstructed point clouds, point clouds with textures, and 3D models.

**Figure 17:**
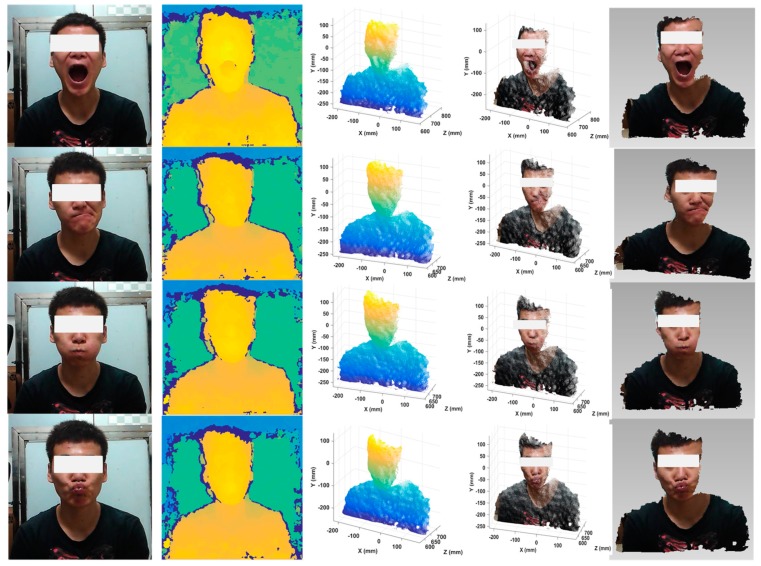
Reconstruction results of dynamic expressions using the proposed system. From left to right: original images, estimated depth maps, reconstructed point clouds, point clouds with textures, and 3D models (mosaic was added over the eyes to protect privacy).

Dynamic objects were also reconstructed using our system. A working fan with a speed of about 30 turns per second and four dynamic human facial expressions were included. Note that the stereo module we used here can work at a frame rate of 60 fps. The single-shot speed of 3D reconstruction mostly depends on the efficiency of stereo rectification and stereo matching, of which the total runtime was controlled to be under 30 ms. In this case, even considering necessary post-processing algorithms and result outputs, the whole pipeline of 3D dynamic reconstruction could be accomplished within a frame rate of not less than 30 fps. To save some space, three frames of the fan data were taken out and put in Figure 16. The results of the four dynamic expressions are displayed in Figure 17. It shows that rather good results were achieved. Even though some regions on the fan blades reflected light to some extent, the corresponding data was recovered accurately. 

## 4. Conclusions

This paper proposes a structured light system based on pseudo-random speckles, which can conduct dense reconstruction with a single shot. The system was constituted by two cameras and a micro-DOE projector. The accuracy of the 3D reconstruction was guaranteed by developing an optimized camera calibration, an accurate epipolar rectification method, and an improved SGM algorithm. Compared with other common 3D vision systems, such as RealSense D435 and Kinect v2, our system performed better. Experiments on complex surfaces and dynamic targets further validated the effectiveness of the proposed system. What should be noted is that the essence of SL is the attachment of textures to the surfaces of objects using optical projectors. It is required that both cameras retrieve similar and textured features. Therefore, it is not suitable for measuring surfaces with high reflectivity variations, which easily produce saturated and low-contrast regions in the captured images. Except for that, this system has good prospects for application and research in the fields of manufacturing, human–computer interaction, robotics, and so on. In future research, the accuracy improvement in the case of short baselines will be deeply researched. 

## Figures and Tables

**Table 1:** Calibration results.

		Parameters Before and After Rectification	
		Original	Results Using Proposed Method
Left camera	Pixel (*H*,*W*)	(1280, 960)	(1280, 960)
	Pixel (*f_x_*,*f_y_*)	(1146.43, 1146.43)	(1063, 1063)
	Pixel (*u*_0_,*v*_0_)	(644.10, 441.84)	(639.31, 438.73)
	(*k*_1_, *k*_2_, *p*_1_, *p*_2_)	(−0.1402, −0.0226, 0.0019, −0.0006)	(0, 0, 0, 0)
Right camera	Pixel (*H*,*W*)	(1280, 960)	(1280, 960)
	Pixel (*f_x_*,*f_y_*)	(1148.88, 1148.88)	(1063, 1063)
	Pixel (*u*_0_,v_0_)	(664.09, 440.03)	(657.20, 438.73)
	(*k*_1_, *k*_2_, *p*_1_, *p*_2_)	(−0.1542, 0.0559, −0.0008, −0.0004)	(0, 0, 0, 0)
*om*		(−0.0095, −0.0025, −0.0019)^T^	(0, 0, 0)^T^
*T* (mm)		(49.95, 0.2490, 0.3229)^T^	(49.97, 0, 0)^T^

Note: the superscript T stands for the transpose operation.

**Table 2:** Accuracy comparison between SGM and improved SGM.

Measurement Distance (mm)		400	600	800	1000
Plane fitting errors (mm)	SGM	0.645	1.905	3.617	5.532
	Our method	0.553	1.462	2.432	3.429
	Difference	↓14.3%	↓23.3%	↓32.8%	↓38.0%
Sphere fitting errors (mm)	SGM	0.669	1.654	2.724	4.988
	Our method	0.465	1.064	1.974	2.701
	Difference	↓30.4%	↓35.7%	↓27.5%	↓44.5%

## Appendix A

The detailed procedures to evaluate the texture richness are provided in this section. The image texture reflects the changes in image color or gray levels, which can generally be evaluated using the gradient distribution of local grayscale values. A small gradient value means that the grayscale changes smoothly, which denotes a poor texture. On the contrary, a large gradient value means that the local grayscale changes distinctly, which denotes a rich texture. Therefore, first, the gradient of each pixel should be calculated, as follows:

(A1) $$\nabla I(u,v)={\left[G_{u},G_{v}\right]}^{T}={\left[\frac{\partial I}{\partial u},\frac{\partial I}{\partial v}\right]}^{T}.$$

Here ∇I(u,v) represents the gradient at pixel ${\left(u,v\right)}^{T}$, where $G_{u}$ and $G_{v}$ denote the gradients in the *u* and *v* directions, respectively. To take both directions into consideration, the gradient map G(u,v) is defined as $G(u,v)=\left\|\nabla I(u,v)\right\|=\sqrt{G_{u}^{2}+G_{v}^{2}}$. In our system, local features within a certain window were taken as the primitives to be matched; therefore, the texture richness should be considered within a predefined window. For that, the gradient map G was convolved using an all--one kernel κ:

(A2) $$G^{\prime }(u,v)=\sum_{i=0,j=0}^{i=m-1,j=n-1}G(u+i-m/2,v+j-n/2)*(\kappa (i,j)/(m\times n)).$$

Here κ(m,n) is an all-one kernel of size m×n. Note that m and n should be odd numbers. $G^{\prime }$ is the windowed gradient map. In this way, the texture richness can be formulated as a sgnfunction:

(A3) $$T(u,v)=\mathrm{sgn}(G^{\prime }(u,v)-\delta \times \delta ).$$

In Equation (A3), δ is the threshold to define rich textures. Generally, δ is set to be an integer no less than 4. If $G^{\prime }(u,v)>\delta^{2}$, then T(u,v)=1, which means the texture of pixel (u,v) is rich; otherwise, T(u,v)=0, which denotes a poor texture at pixel (u,v).
